# Retrograde Autologous Talar Osteocancellous Bone Grafting for the Treatment of Osteochondral Lesions of the Talus: A Technical Note

**DOI:** 10.3390/jcm12103431

**Published:** 2023-05-12

**Authors:** Takuji Yokoe, Takuya Tajima, Nami Yamaguchi, Yudai Morita, Etsuo Chosa

**Affiliations:** Division of Orthopaedic Surgery, Department of Medicine of Sensory and Motor Organs, Faculty of Medicine, University of Miyazaki, 5200 Kihara, Kiyotake, Miyazaki 889-1692, Japan

**Keywords:** osteochondral lesion, talus, surgery, retrograde grafting, minimally invasive

## Abstract

Osteochondral lesions of the talus (OLT) are common injuries in young athletes. Various kinds of surgical procedures are available for orthopaedic surgeons, but which surgical technique is the best remains controversial. Many surgical procedures require malleolar osteotomy to obtain appropriate surgical exposure to the OLT because of the anatomic characteristics of the ankle joint. However, malleolar osteotomy is invasive and has a potential risk of complications, such as tibial chondral damage and pseudoarthrosis. This article aims to introduce a novel surgical procedure for the treatment of OLTs: retrograde autologous talar osteocancellous bone grafting without the need for osteotomy and harvesting a graft from anywhere other than the talus. First, an arthroscopic evaluation is performed to verify the location, size, and cartilage quality of the OLT as well as concomitant lesions. After confirming the position of the guide pin using a guide device arthroscopically, a talar osteocancellous bone plug is harvested using a coring reamer. The OLT of the harvested talar bone plug is removed, and under arthroscopy, the talar osteocancellous bone plug is retrogradely inserted into the talar bone tunnel. To stabilize the implanted bone plug, one or two bioabsorbable pins are inserted from the lateral wall of the talus while applying counterforce to the articular surface of the bone plug. The present surgical technique can minimally invasively address the OLT without the need for malleolar osteotomy and harvesting a graft from the knee joint or iliac bone.

## 1. Introduction

Osteochondral lesion of the talus (OLT) usually occurs in young athletes. A number of surgical treatments for OLTs have been reported, including bone marrow stimulation (BMS), autologous osteochondral transplantation, fixation of the osteochondral fragment, osteochondral allograft transplantation, autologous matrix-induced chondrogenesis (AMIC), and autologous chondrocyte implantation [1,2,3,4,5,6]. However, at present, which surgical procedure is the best remains controversial [2]. Traditionally, BMS has been the most commonly performed procedure for OLTs [1,7,8]. However, lesion size is a well-known predictor of clinical results following BMS [7,9], and a recent systematic review reported that BMS may be best considered for OLTs <10.2 mm in diameter and/or 107.4 mm^2^ in area [10]. Furthermore, several studies have shown that about one-third of lesions treated by BMS showed incomplete healing according to a second-look arthroscopic evaluation [11,12]. Rikken et al. also reported that progressive degeneration of the cartilage was found in 28% of the patients at a mean of 13 years after BMS [8]. Therefore, to improve the clinical outcomes following BMS, several concomitant procedures have been introduced with promising clinical outcomes. Favorable surgical outcomes up to 5 years following AMIC have been reported [13,14,15,16]. However, the majority of the studies (13/14 studies) were of a low level of evidence, suggesting the necessity of further prospective studies with a control group clarifying the efficacy of the AMIC. Additionally, at present, AMIC can be performed for OLTs in limited countries. In addition to the AMIC technique, adjuvant biologic therapies, including plate-rich plasma, concentrated bone marrow aspirate, adipose-derived stem cells, and hyaluronic acid, have been reported [7,17,18,19,20]. However, there is a lack of consensus on which biologics can accelerate the healing of OLTs and provide better clinical outcomes [18].

For large OLTs, osteochondral autograft transplantation is a procedure with favorable clinical results [14,21]. However, there is some concern about the associated risk, including donor-site morbidity and malleolar osteotomy [22,23,24]. Moreover, inherently different characteristics between the knee and ankle cartilage, such as stiffness, proteoglycan synthesis and turn-over, and response to injury, have been reported [25,26,27]. Takao et al. reported retrograde transplantation of a cancellous bone plug from the iliac crest for the treatment of a large OLT with a good short-term result [28]. However, in this surgical procedure, the debrided space of the lesion is filled with iliac cancellous bone, the characteristics of which are different from those of talar cancellous bone and carry a risk of donor-site morbidity. The aim of the present technical note is to report a novel surgical treatment for OLTs: retrograde autologous talar osteocancellous bone grafting without the need to harvest a graft from sites other than the talus.

## 2. Surgical Procedures

### 2.1. Preoperative Evaluation

The diagnosis of OLT is confirmed by consideration of the patient’s history, findings of the physical examination (tenderness to the lesion and ankle range of motion), and diagnostic imaging. The presence of chronic lateral ankle instability is also assessed by physical examinations (anterior drawer test, reverse anterolateral drawer test, and inversion stress test), stress radiography, and ultrasonography. Before surgery, computed tomography is routinely performed to evaluate the location and size of the lesion. Magnetic resonance imaging is also performed to evaluate the quality of the cartilage, subchondral edema, and cyst. Surgery is considered when a patient’s symptoms do not improve after 4–6 months of conservative treatment, such as protected weightbearing, physical therapy, and medication.

### 2.2. Surgical Techniques

**Figure 1:**
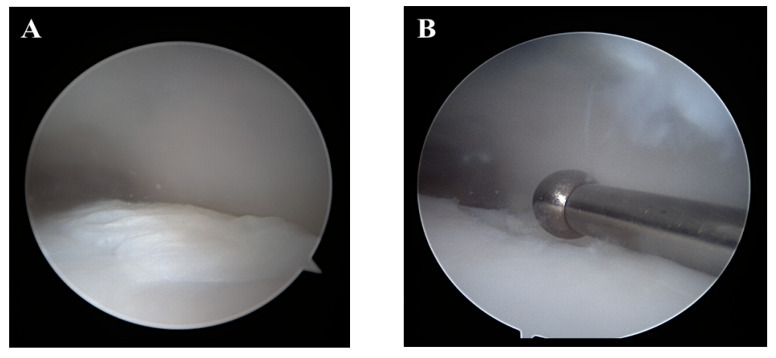
Arthroscopic images of osteochondral lesion of the talus. Right ankle. (**A**) An osteochondral lesion of the talus shows damaged cartilage. (**B**) The position of the guide pin is confirmed using a guide device.

**Figure 2:**
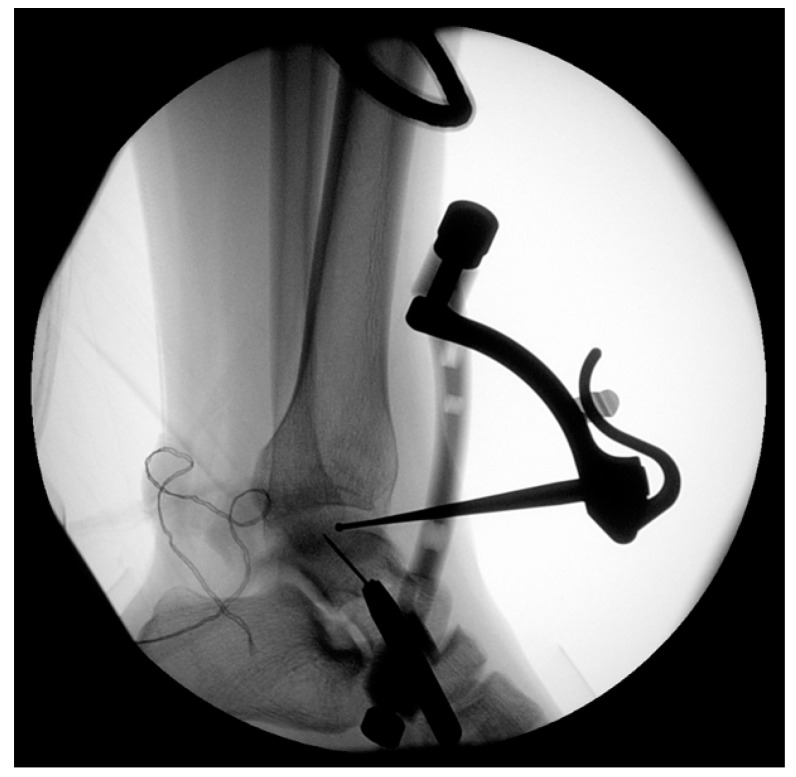
Intraoperative radiographic images. The direction and position of the guide pin is confirmed using a guide device. Lateral view of the right ankle.

Surgery was performed with the patient in the supine position on the operating table under general or spinal anesthesia. A bump is placed under the ipsilateral hip to enable easy access to the lateral side of the foot, and the hip is flexed to 45° in a leg holder while applying an ankle distraction device. A tourniquet is inflated over the ipsilateral thigh, depending on the surgeon’s preference. First, the diagnostic arthroscopic evaluation is performed through standard anteromedial and anterolateral portals. Concomitant pathologies, such as lateral ankle instability, are identified and should be treated. An approximately 30-mm transverse incision is made over the sinus tarsi, and the entry point of the Kirshner wire to the lateral aspect of the talus is confirmed under fluoroscopy. The talar insertion of the anterior talofibular ligament should be carefully exposed and prevented from an iatrogenic injury. A Kirshner wire of 1.0–1.2 mm in diameter is inserted toward the lesion using a guide device (New GPS Targeting Drill Guide, Arthrex, Naples, FL, USA), under fluoroscopy and arthroscopy (Figure 1 and Figure 2).

**Figure 3:**
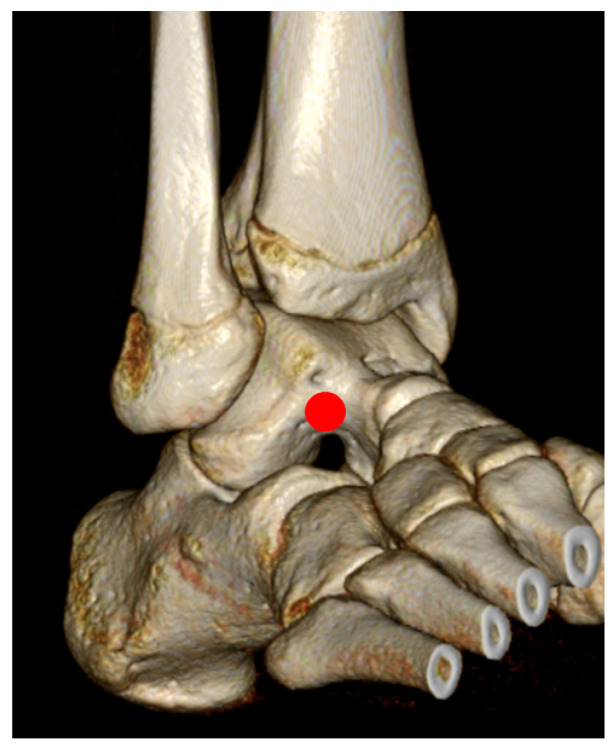
Picture showing the entry-point (red circle) of the coring reamer (Coring reamer, Arthrex, Naples, FL, USA). The talar insertion of the anterior talofibular ligament should be carefully exposed and avoided from an iatrogenic injury.

**Figure 4:**
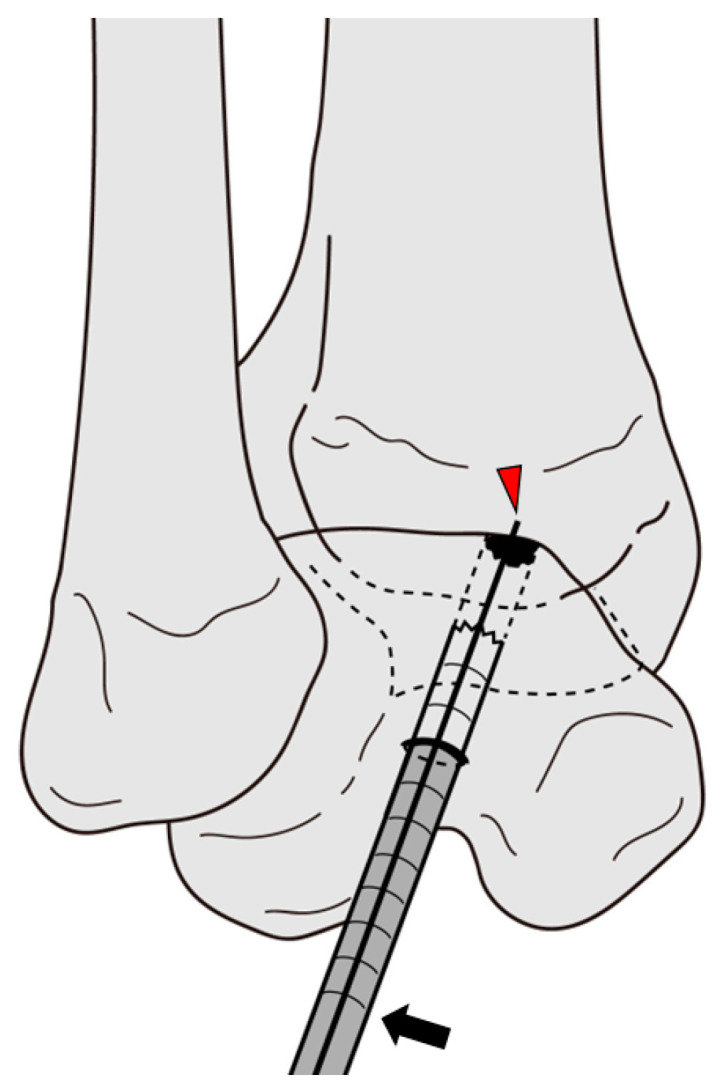
Picture showing harvesting of the talar osteocancellous bone plug using a coring reamer after inserting a guide wire with a guide device (New GPS Targeting Drill Guide, Arthrex, Naples, FL, USA). Black arrow, coring reamer; Red arrowhead, Kirshner wire.

**Figure 5:**
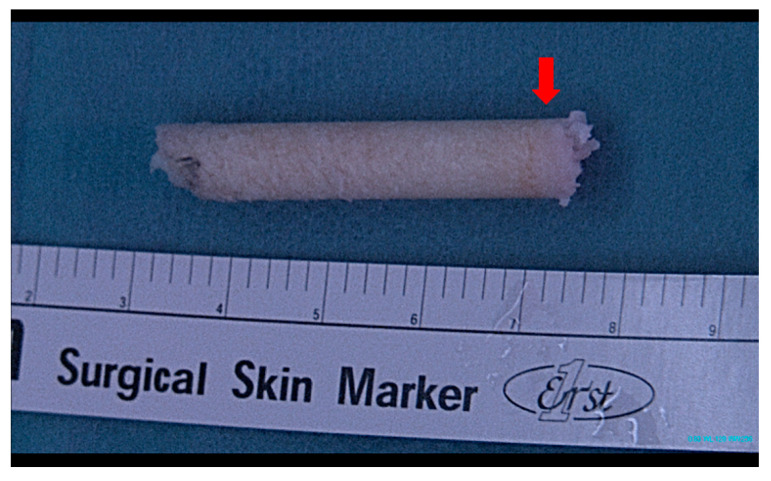
Harvested talar osteocancellous bone plug. The red arrow shows the osteochondral lesion of the talus (articular surface of the bone plug). The lesion of the bone plug is removed using a small bone rongeur before inserting the bone plug into the talar bone tunnel.

A talar osteocancellous bone plug (8.0 or 9.0 mm in diameter) is harvested from the exposed lateral side of the talus under fluoroscopy and arthroscopy using a coring reamer (Coring reamer, Arthrex, Naples, FL, USA) (Figure 3, Figure 4 and Figure 5).

**Figure 6:**
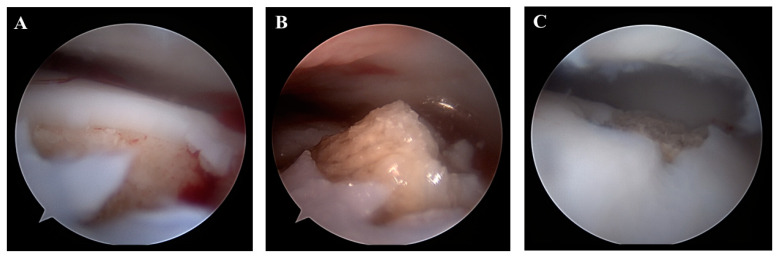
The talar osteocancellous bone plug is retrogradely inserted into the talar bone tunnel. Right ankle: (**A**) before inserting the bone plug; (**B**) after inserting the bone plug; (**C**) the prominent cancellous bone is smoothened using an arthroscopic shaver.

**Figure 7:**
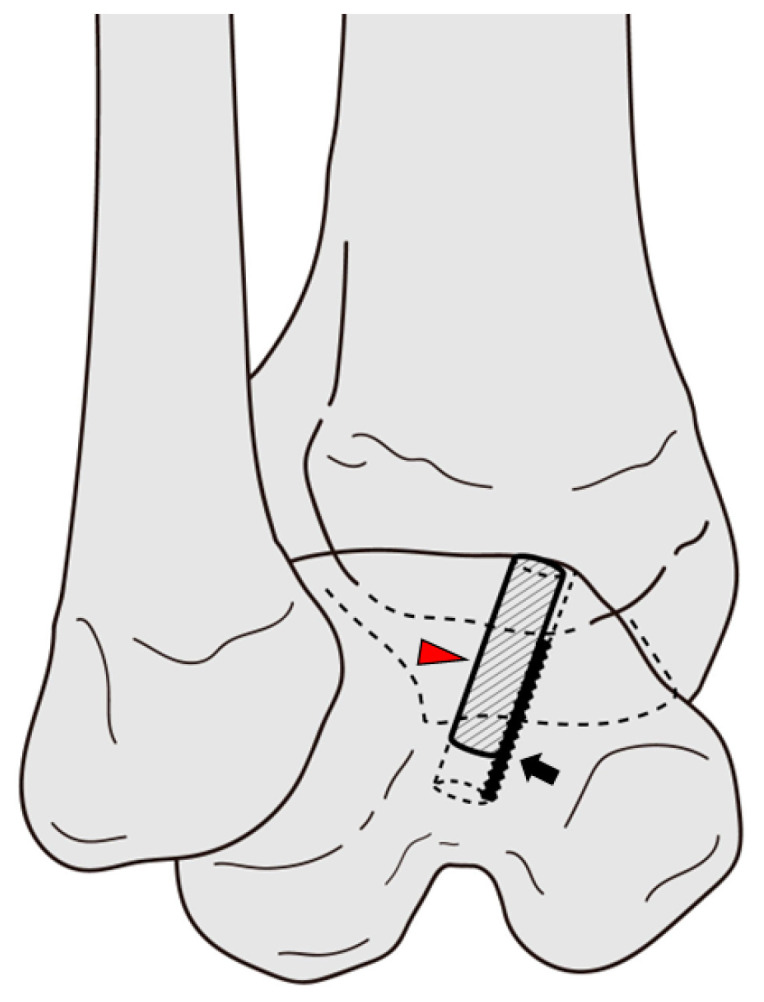
Picture showing stabilization of the inserted talar osteocancellous bone plug with bioabsorbable pin. Red arrowhead, talar osteocancellous bone plug; Black arrow, bioabsorbable pin.

The subchondral lesion is additionally debrided with a curette or arthroscopic shaver via fluoroscopy if needed. The lesion is removed from the talar osteocancellous bone plug, after which the bone plug is retrogradely inserted into the created bone-tunnel arthroscopically (Figure 6A,B). To stabilize the implanted bone plug, 1 or 2 bioabsorbable pins (2.0 to 3.0 mm in diameter) are inserted between the bone plug and the surrounding talar tunnel from the lateral wall of the talus while applying compression force to the articular surface of the bone plug with an elevator with raspatories. Via arthroscopy, using an arthroscopic shaver, the articular surface of the cancellous bone plug is smoothed to avoid making a gap between the adjacent native articular cartilage and in order not to impinge with the tibial plafond (Figure 6C and Figure 7).

**Figure 8:**
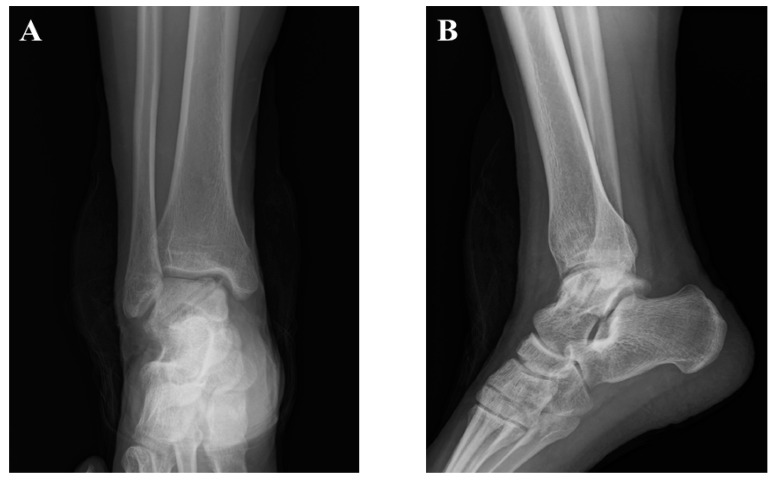
Post-operative radiographic images. (**A**) Antero-posterior view; (**B**) lateral view of the right ankle.

Artificial bone or allograft is used to fill the void of the lateral surface of the talus, depending on the surgeon’s preference. Surgical incisions are closed in a standard fashion. Post-operative radiographic images are shown in Figure 8. Surgeons should take care not to cause iatrogenic damage to the tibial articular surface throughout this surgical technique.

### 2.3. Postoperative Rehabilitation

**Figure 9:**
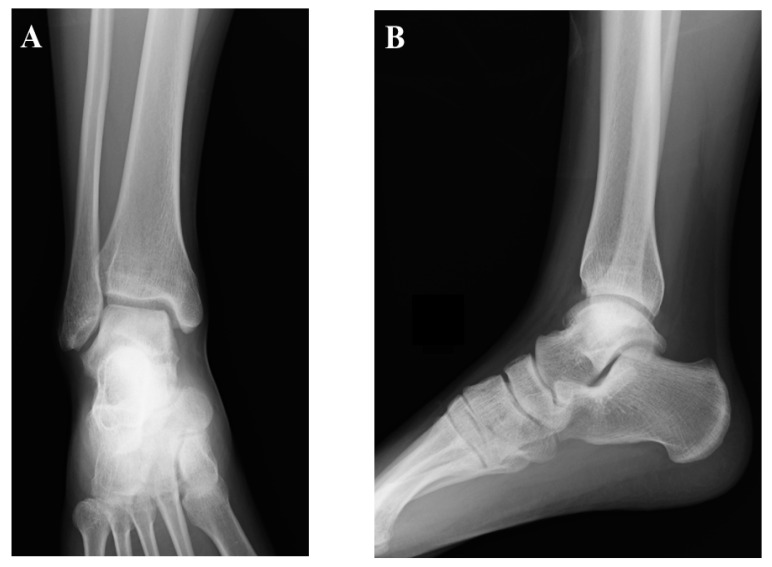
Radiographic findings at 12 months after surgery. (**A**) Antero-posterior view; (**B**) lateral view of the right ankle.

The ankle is immobilized with a below-knee splint for two weeks after surgery, with no-weight bearing sustained for four to six weeks. Ankle range of motion (ROM) exercises are initiated at two weeks post-operatively. Weight bearing is gradually increased, and full weight bearing is initiated at 8–10 weeks after surgery. Running is allowed at four months after surgery, followed by a return to agile activities and sports at six to eight months after surgery. The radiographic findings at 12 months after surgery are shown in Figure 9.

The advantages and disadvantages of the present surgical procedure are summarized in Table 1.

## 3. Discussion

The surgical technique described here may enable treating medial-sided OLTs efficiently and as lowly invasively as possible compared with other procedures. First, access to the OLT does not require malleolar osteotomy due to retrograde grafting. Therefore, complications associated with malleolar osteotomy, such as nonunion and damage to the tibial articular cartilage, can be avoided [22,23,24]. Second, harvesting a graft from a site other than the talus is not needed, as an intact (healthy) talar osteocancellous bone plug can be applied to the lesion. Therefore, donor-site morbidities, such as persistent pain, knee stiffness, and patellofemoral disturbance, do not occur [29,30]. A systematic review reported that the rate of donor-site morbidity after knee-to-ankle mosaicplasty was 16.9% [31].

Takao et al. harvested an 8.5-mm cancellous bone plug from an iliac crest for retrograde grafting [28]. Autologous bone harvesting from the iliac crest is a frequently performed procedure in surgery for nonunion, arthroplasty, and spinal fusion. It was reported that approximately 500,000 bone grafts were harvested annually in the United States [32]. However, autologous bone graft harvesting from the iliac crest is not without complications. A systematic review described that the rate of morbidity from harvesting from the anterior iliac crest was 18.9% (603/3180 patients), and chronic pain (6.4%) was the most frequent complication, followed by sensory disturbance (5.2%) and infection (1.8%) [33]. Additionally, pain at the donor site will hinder the post-operative rehabilitation in the early phase after surgery. Therefore, the presented technique may be less invasive and safer than the procedure described by Takao et al. Furthermore, there are likely qualitative and quantitative differences between the talar and iliac cancellous bone, although no study has clarified this issue. Sagi et al. reported that, by evaluating the messenger RNA expression profiles, graft material from the medullary canal of the femur or tibia contained more growth factors and stem cell markers related to the osteogenic cascade than that from the iliac crest [34]. Empirically, the structure of the talar cancellous bone is denser and sturdier than that of the iliac crest. The talar body reportedly has a pronounced subchondral lamella, enabling it to withstand great compressive force during walking and running [35,36,37]. Therefore, it makes sense for the debrided subchondral space of the OLT to be replaced not with iliac cancellous but with talar cancellous bone. Further studies will be needed to compare the differences in the biological and biomechanical qualities between the talar and iliac bone to investigate which graft is most appropriate as a graft material for the treatment of OLTs.

The present technique may be associated with some risks and complications, including talar neck fracture and avascular necrosis of the talus due to creating a relatively large bone hole. Fortunately, we have never encountered these complications. Additionally, there is a risk of damaging the talar insertion of the anterior talofibular ligament, and careful exposure of the entry point of the guide wire should therefore be performed in order to avoid these complications. In this surgical technique, the hyaline cartilage cannot be restored. Although the application of collagen membrane to the OLT is not allowed in the author’s country, application of the collagen membrane or orthopaedic biologics combined with the presented surgical procedure may be a promising viable option. According to the recommendations by the International Consensus Group on Cartilage Repair of the Ankle [38], the use of additional scaffolds may be considered for OLTs with a lesion size of >1 cm^2^. However, several studies have reported that application of the collagen membrane to the BMS or autologous bone grafting did not provide better clinical outcomes [39,40]. Therefore, the efficacy of the present surgical procedure augmented with collagen membrane or biologics needs to be assessed in future studies. Finally, clinical outcomes after the current procedure remain unclear. We are planning to prospectively assess the patients undergoing this technique.

In conclusion, we presented a novel technique for retrograde talar osteocancellous bone grafting for the surgical treatment of OLT. This surgical technique may be a minimally invasive, effective, and logical surgical procedure. Future studies will be needed to verify its clinical effectiveness and safety as a surgical procedure of OLTs.

## Figures and Tables

**Table 1:** Advantages and disadvantages of retrograde talar osteocancellous bone grafting.

**Advantages**
1. Healthy talar cancellous bone can be used as an autograft.
2. No need for malleolar osteotomy.
3. No need to harvest an autograft (iliac crest, knee).
**Disadvantages**
1. The hyaline cartilage cannot be restored.
2. There is a risk of iatrogenic damage to the talar insertion of the ATFL.
3. There is a risk of iatrogenic damage to the tibial articular cartilage.

## Data Availability

The data presented in the present study are available on request from the corresponding author.
